# The Corruption of Charity

**Published:** 1920-01-10

**Authors:** C. E. Hoar

**Affiliations:** Chairman of the Kent County Ophthalmic Hospital. Maidstone


### THE CORRUPTION OF CHARITY.

*To the Editor of* The Hospital.

Sir,—In your issue of December 20, 1919, you comment on the number of weekly subscribers to the Kent County Ophthalmic Hospital, and the free in-patient and out-patient treatment to every weekly subscriber, "whatever his wages," and to the members of his family, if wholly dependent on him. There is, however, a qualifying clause in the regulations, *viz.*, "The above only applies to members whose individual wages are not more than £200 per annum, exclusive of war bonus." I shall be glad if you will publish this correction.—Yours faithfully,

C. E. Hoar, M.D.,
Chairman of the Kent County Ophthalmic Hospital.

Maidstone, December 30, 1919.

